# AGING WELL: OLDER ADULTS AND DATA MAPPING

**DOI:** 10.1093/geroni/igad104.0712

**Published:** 2023-12-21

**Authors:** Susan Silberman

**Affiliations:** National Council on Aging, Arlington, Virginia, United States

## Abstract

This presentation will feature how the National Council on Aging (NCOA) has advanced a program of work on data mapping and visualization. It will explain how this work is part of NCOA’s mission to improve the lives of millions of older adults, especially those who are struggling. Guided by the idea that how we age should not be determined by gender, color, sexuality, income, or zip code, the mapping tools display area-level data to highlight different aspects of the lives of low-income older adults. This presentation will describe two of NCOA’s mapping tools and how they can influence policy and advocacy efforts toward aging equity at the local, regional, state, and national levels. The first tool, released in 2021, displays, in part, area-level access to smartphones and high-speed internet among low-income older adults. This map makes visible the invisible by demonstrating low rates of access even in geo-spatial areas with overall high levels of connectivity. The second tool, to be released in the third quarter of 2023, presents sub-state level data to identify gaps between area-level eligibility for each of three essential benefit programs [Supplemental Nutrition Assistance Program (SNAP)], Supplemental Security Income (SSI), and Medicare Savings Program (MSP)] and area-level enrollment rates. This map demonstrates how socio-spatial positions influence access to these national benefits, and how maps can be used to identify regions and communities to prioritize as part of strategic efforts to improve benefit access and promote aging equity.

